# Supplementary material for: Dopamine D1 Receptor in Cancer
Source: Cancers (Basel). 2020 Nov 2;12(11):3232. doi: 10.3390/cancers12113232 (PMC7693420; doi:10.3390/cancers12113232)
Supplement: Supplementary file 1 [file cancers-12-03232-s001.pdf]

Supplementary Materials: Dopamine D1 Receptor in Cancer

Paweł Sobczuk, Michał Łomiak and Agnieszka Cudnoch-Jędrzejewska

Table S1. Summary of current data about mRNA and protein expression of type 1 dopamine receptor. Name of cell line or type of examined material is given in brackets.

Cancer Type	D1r Expression	Ref.
Acute T cell leukemia	- protein (Jurkat cells) - present	[107], [110]
Astrocytoma	protein (D384) – present	[167]
Lymphocytes B malignancies	-mRNA (DOHH2, H929, KMS11, K1106, K422, LILA OZ, PRI, RAMOS, REC-1) – present or overexpression	[168]
Cholangiocarcinoma	- mRNA (Mz-ChA-1) - overexpression<br>- mRNA (HuCCT-1, CCLP-1) downregulation<br>- mRNA (SG231) – present	[169]
Colon cancer	- mRNA (HCT116, HT29, RKOp, RKOr1) overexpression	[58,170]
Ependydoma	-protein (patients` samples) – overexpression in 30% cases	[171]
Gist	- protein (patients` samples) - overexpression	[171]
Glioma	- protein (patients samples) - overexpression	[103]
Hepatocellular carcinoma	- mRNA (Hep3B, patients samples) – upregulation	[58], [172]
Melanoma	- protein (patients` samples) – overexpression in less than 25% cases<br>- lack of mRNA for D1 receptor in cell lines derived from melanoma metastases	[171,173]
Myelogenous leukemia	- protein (K562) - present	[171]
Neuroblastoma	-protein (NS20Y)<br>- mRNA (SK-N-MC, SKNSH) - upregulation	[58,174,175]
Osteosarcoma	- protein (OS732) - present	[55]
Ovarian cancer	- protein (patients` samples) – overexpression in 50% cases	[171], [23,126]
Pancreatic cancer	- protein (SW1990, patient-derived xenografts) - upregulation	[116]
Pancreatic neuroendocrine tumor	mRNA (BON) - present	[176]
Pheochromocytoma	- protein (patients` samples) – overexpression in less than 25% cases	[171]
Renal cell cancer	- protein (patients` samples) – overexpression in 50% cases	[171]
Retinoblastoma	-protein (WERI 27)	[175]
Synovial sarcoma	-protein (patents` samples)- overexpression in 21% cases	[171]
Small cell lung carcinoma	- protein (patients` samples, patients` derived cell lines, H69) - overexpression	[113]

Table S2. Dopamine D1 receptor agonists and antagonists.

Name	Other information
**Agonists**	
6-Br-APB	selective D1 receptor agonist<br>90-fold selectivity for D_1 over D_2
A-68930	selective D1 receptor agonist
A-77636	selective D1 receptor agonist
A86929	selective dopamine D1 receptor agonist

	14-fold selectivity for D_1-like receptors over D_2
dihydrexidine	selective dopamine D1 receptor agonist<br>10-fold selectivity for D_1-like receptors over D_2
dinapsoline	selective dopamine D1 receptor agonist<br>5-fold selectivity for D_1-like receptors over D_2
doxanthrine	dopamine D1 receptor and D2 receptor agonist<br>168-fold greater selectivity for D1 over D2 receptors
fenoldopam	highly selective peripheral D_1 receptor partial agonist
PF-06649751	dopamine D1 and D5 receptors partial agonist
SKF89145	selective D1 receptor agonist
SKF89626	selective D1 receptor agonist
SKF38393	very high selectivity for D_1 with negligible affinity for any other receptor
SKF81297	dopamine D1 and D5 receptors agonist<br>200-fold selectivity for D_1 over any other receptor
SKF82958	dopamine D1 and D5 receptors agonist<br>57-fold selectivity for D_1 over D_2
stepholidine	D1 agonist and D2 antagonist
**Antagonists**	
ecipopam (SCH39166)	D1 and D5 receptors antagonist
SCH23390	D1 receptor agonist with minimal effect of D2 receptor

© 2020 by the authors. Licensee MDPI, Basel, Switzerland. This article is an open access article distributed under the terms and conditions of the Creative Commons Attribution (CC BY) license (http://creativecommons.org/licenses/by/4.0/).
